# Successful Treatment of Eosinophilic Granulomatosis With Polyangiitis: A Case of Refractory Peripheral Neuropathy and Comorbid Chronic Progressive Pulmonary Aspergillosis Treated With Mepolizumab

**DOI:** 10.7759/cureus.52192

**Published:** 2024-01-12

**Authors:** Ryu Sekiya, Tomoyuki Soma, Kazuyuki Nakagome, Makoto Nagata

**Affiliations:** 1 Department of Respiratory Medicine, Allergy Center, Saitama Medical University, Saitama, JPN; 2 Preventive Medical Center, Saitama Medical University Hospital, Saitama, JPN

**Keywords:** chronic pulmonary aspergillosis, antineutrophil cytoplasmic antibody (anca) associated vasculitis (aav), neurological manifestation, mepolizumab, eosinophilic granulomatosis with polyangiitis (egpa)

## Abstract

Eosinophilic granulomatosis with polyangiitis (EGPA) is a systemic necrotizing vasculitis accompanied by granulomas and eosinophilic inflammation, exhibiting marked peripheral blood eosinophiliaandasthma. Neuropathy is a difficult-to-treat common manifestation that frequently remains after achieving clinical remission with current therapy in a subpopulation of patients with EGPA with or without life-threatening organ involvement. Refractory neuropathy regularly reduces the quality of life and requires glucocorticoids (GCs) and/or immunosuppressants for a long time. Long-term immunosuppressive therapy is a factor associated with a high risk of adverse effects. Mepolizumab, at three times the dose for severe asthma, provides benefits to induce the remission of relapsing or refractory EGPA and to reduce the doses of GC. Here, we present a case of EGPA successfully treated with mepolizumab at the reference dose for severe asthma. In this case, mepolizumab resolved peripheral neuropathy resistant to corticosteroids, immunosuppressants, and intravenous immunoglobulin and contributed to the improvement of comorbid chronic pulmonary aspergillosis during GC dose reduction.

## Introduction

A systemic inflammatory condition known as eosinophilic granulomatosis with polyangiitis (EGPA) was previously known as Churg-Strauss syndrome, showing general symptoms (fever, arthralgia, headache, weight loss, etc.) and multiorgan impairment, such as nasal and paranasal disease, cardiovascular involvement, pulmonary involvement, nephritis, central and peripheral nerve system dysfunction, gastrointestinal disease, and skin involvement [1-4]. Patients with EGPA often have asthma or asthmatic manifestations and marked peripheral blood eosinophilia before EGPA develops [1-4]. Histological findings include interstitial and perivascular necrotizing granulomas, along with the infiltration of many eosinophils into the tissue and vasculitis in small- and medium-size vessels [4-6]. Upon therapy induction, glucocorticoids (GCs) alone are recommended for patients newly diagnosed with EGPA without poor prognosis factors, scoring zero on the Five-Factor Score (FFS) [4], and a combination of GCs and cyclophosphamide (CYC) is recommended for those with ≥1 FFS or other life-threatening manifestations to achieve clinical remission [1,7-11]. During the period of maintenance therapy, low doses of GCs, methotrexate, and azathioprine are recommended to preserve clinical remission [1,4-11].

Notably, most patients necessarily continue GCs and/or immunosuppressants for a long time because EGPA is recognized as a chronic relapsing disease [1,5-11]. Long-term immunosuppressive therapy may lead to a high risk of treatment-related complications [9,10]. In particular, prolonged exposure to GCs is linked to a four-fold increase in the risk of metabolic problems such as weight gain, hyperglycemia, and type 2 diabetes, as well as hypertension (above 30%), bone fractures (around 20%-30%), cataracts (1%-3%), and gastrointestinal conditions (1%-5%) [12]. In addition, short- and long-term GCs used to resolve acute asthma exacerbations and to chronically control asthma conditions increase the risk of acute and chronic complications, the risk of which is increased by greater exposure [13]. In addition, the use of GCs could lead to the progression of underlying health conditions, such as chronic infection and type 2 diabetes [12,13].

Peripheral mononeuropathy multiplex is a major manifestation of EGPA (~50%-90%) [1,7,8]. Contrary to the feasible efficacy of initial conventional therapies on systemic manifestations with life-threatening involvements, such as heart involvement and renal disease, peripheral neuropathy is resistant to these manifestations in approximately half of EGPA patients [1,2,7,8,14]. Among patients with EGPA, persistent neuropathic deficiency can necessitate the restriction of their activities of daily living and lead to remarkable stress because of low muscle strength and pain in the extremities [5,6]. These patients also need to continue immunosuppressive therapies for years, resulting in an increase in the risk of infectious disease because of their immunocompromised state [12,13].

Mepolizumab, a humanized monoclonal anti-interleukin-5 antibody, was recently approved for treating EGPA following severe eosinophilic asthma [9,10]. Mepolizumab has remarkable benefits for severe eosinophilic asthma and has achieved a feasible remission rate and decreased GC dose in certain patients with relapsing or refractory EGPA [15]. The American College of Rheumatology (ACR)/Vasculitis Foundation and the European Alliance of Associations for Rheumatology (EULAR) have recommended mepolizumab as an induction therapy for remission in patients with relapsing or refractory EGPA without active organ-threatening or life-threatening disease [9,10].

We report a case in which the reference dose of mepolizumab for severe eosinophilic asthma successfully resolved refractory mononeuropathy multiplex involved in EGPA resistance to a moderate dose of oral corticosteroid (OCS) after remission and contributed to the improvement of comorbid pulmonary aspergillosis treated with antifungal medicine.

## Case presentation

**Figure 1:**
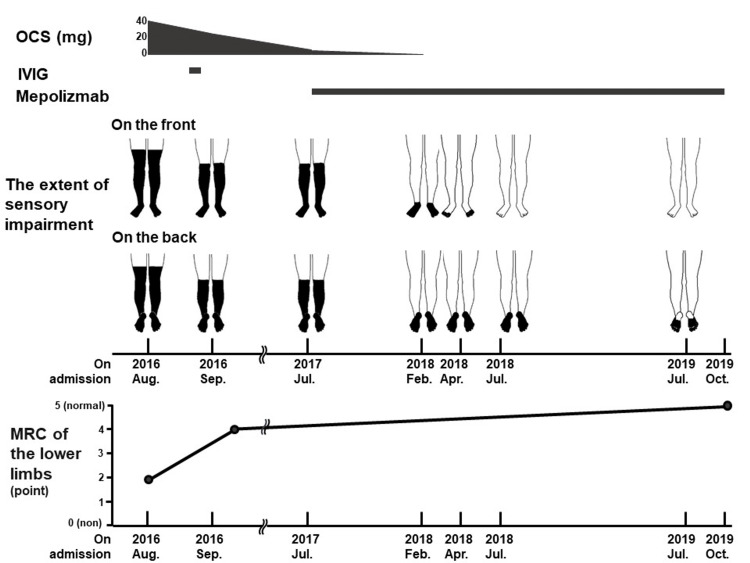
Treatment and clinical course. The patient initially received OCS and additional IVIG therapy. The amplitude and extent of sensory impairment in the lower extremities remained unresolved. This impairment mostly disappeared after the initiation of mepolizumab. The MRC grade of movement disturbance in the lower extremities improved after starting OCS and mepolizumab. OCS: oral corticosteroid; IVIG: intravenous immunoglobulin; MRC: Medical Research Council.

A 73-year-old man with peripheral blood eosinophilia, numbness, and purpura in the legs and preexisting asthma was admitted to our hospital. He had hypoesthesia in his legs and bilateral ankle dorsiflexion disturbance (Medical Research Council (MRC) grade 2; 0 (no contraction) to 5 (normal power)) (Figure 1).

**Figure 2:**
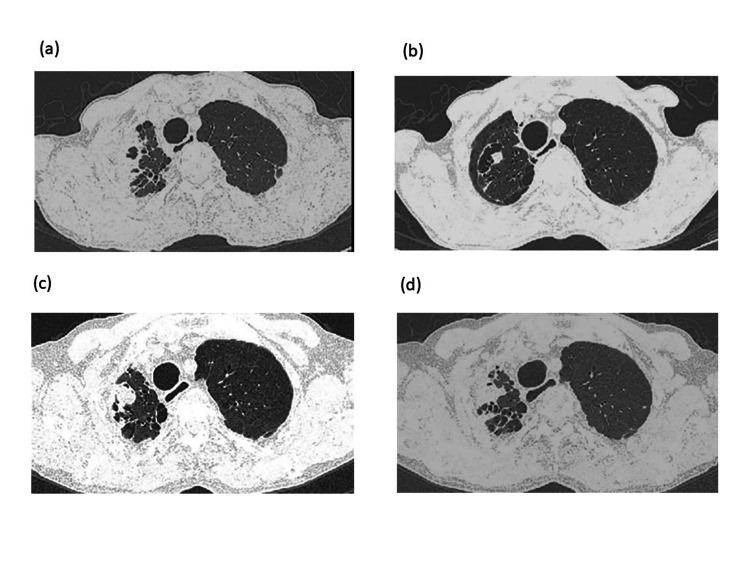
Chest CT. The CT scan reveals a cavity and pleural thickening of the right upper lung on day 1 (a), the disappearance of pleural thickening and residual cavity with a fungus ball lesion one month after receiving OCS (b), fungus ball lesion progression of the right lung while tapering OCS (c), and collapsed fungus ball lesion during mepolizumab administration (d). CT: computed tomography; OCS: oral corticosteroids.

Blood examination results were as follows: leukocytes, 17,160/µL; eosinophil counts, 11,497/µL (67%); C-reactive protein (CRP), 5.7 mg/dL; immunoglobulin E (IgE), 3162 IU/mL; *Aspergillus* galactomannan antigen, 1.1 (cutoff <0.5); positivity for *Aspergillus* IgG antibody; and Japanese cedar pollen-specific IgE, 10.0 UA/mL. Notably, the myeloperoxidase antineutrophil cytoplasmic antibody (MPO-ANCA) was not detected. Chest computed tomography (CT) revealed a cavity with a thickened wall in the right upper lobes and right pleural thickness on day 1 (Figure 2a).

**Figure 3:**
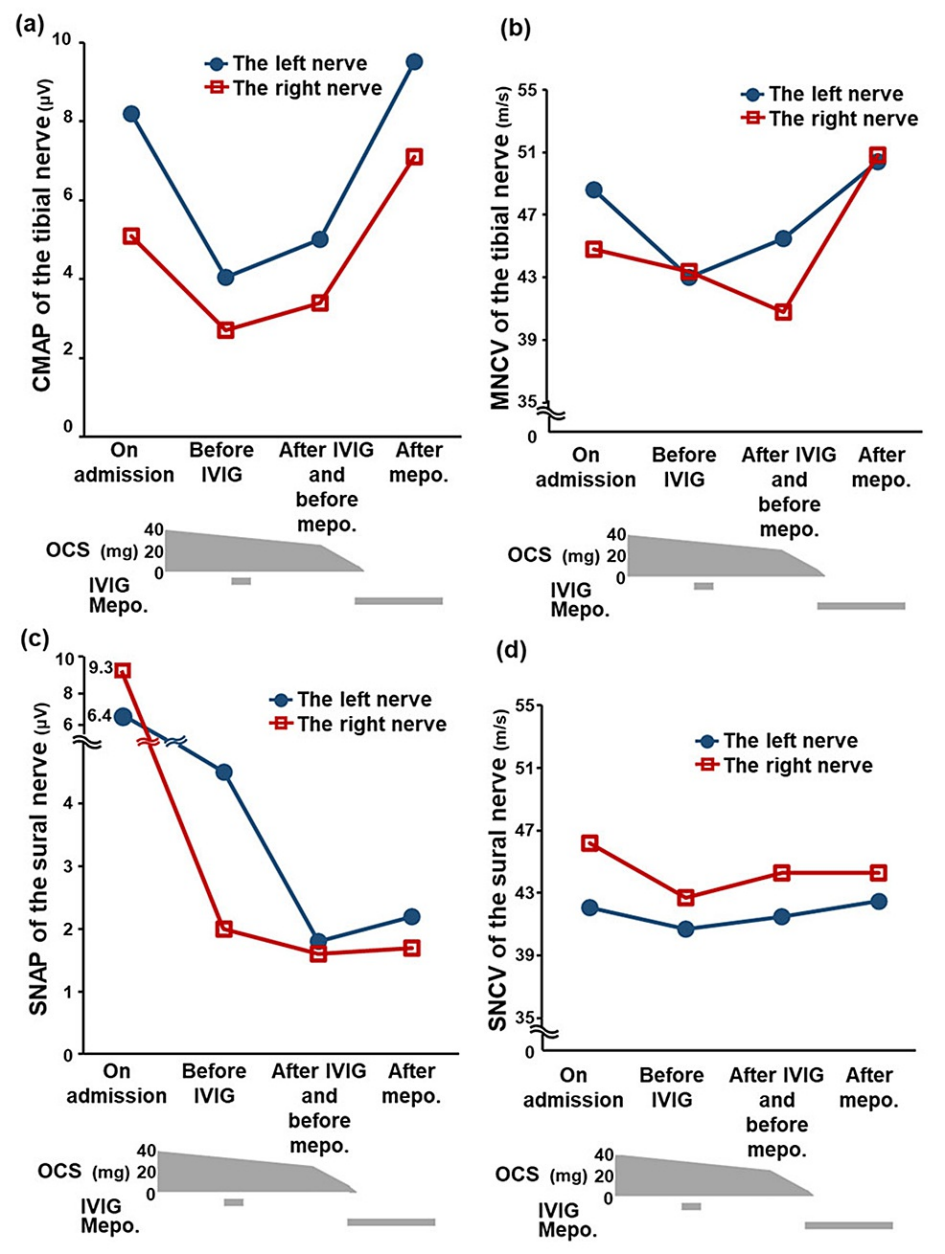
Nerve conduction study changes. CMAP (a) and MNCV (b) in tibial nerves and SNAP (c) and SNCV (d) in sural nerves improved during OCS, IVIG, and Mepo. therapy. Closed circle: left nerve; open square: right nerve. CMAP: compound motor action potential; MNCV: motor nerve conduction velocity; SNAP: sensory nerve action potential; SNCV: sensory nerve conduction velocity; OCS: oral corticosteroid; IVIG: intravenous immunoglobulin; Mepo.: mepolizumab.

Bronchial alveolar lavage fluids exhibited an elevated eosinophil percentage of 48% (reference range: ≤1%). Histological examination of purpuric lesions on the left leg revealed marked infiltration of eosinophils and lymphocytes into the edematous dermis, particularly around the vascular area. A nerve conduction study (NCS) revealed a modest reduction in the compound motor action potential (CMAP; reference range: 5.0-21.4 mV) and a marginal reduction in the motor nerve conduction velocity (MNCV; 41-55 m/s) in the bilateral tibial nerves, along with a modest decrease in the sensory nerve action potential (SNAP; 3.1-15.1 µV) and a marginal decline in the sensory nerve conduction velocity (42.7-51.7 m/s) in the sural nerve (Figure 3a-d). Accordingly, EGPA and chronic pulmonary aspergillosis (CPA) were diagnosed. The patient’s initial Birmingham Vasculitis Activity Score (BVAS) was 23 points.

Oral prednisolone at 0.8 mg/kg/day was initiated on day 12 and tapered one month later. Moreover, 400 mg of voriconazole was orally administered. Blood eosinophils and CRP levels immediately decreased to 0/µL and 0 mg/dL, respectively. However, sensory symptoms remained severe, and CMAP, MNCV, and SNAP rapidly deteriorated (Figure 3a-c). After intravenous immunoglobulin (IVIG) administration, ankle dorsiflexion deterioration improved (Figure 1). Meanwhile, dense shadows around the cavity in the right upper lobe were reduced, but a nodule inside the cavity remained on chest CT (Figure 2b). Voriconazole was switched to itraconazole at 200 mg a day because of a disturbance in liver function after three weeks. He was discharged on day 62 as his BVAS decreased to six points.

As a maintenance treatment, prednisolone was reduced to 5 mg/day over nine months. Contrary to the improvement in his laboratory data, he experienced persistent hypoesthesia and paresthesia in the legs during the course of the study (Figure 1). Additionally, the fungus ball lesion enlarged on chest CT after nine months despite continuous treatment with itraconazole (Figure 2c). Considering the additive effect of OCS on peripheral neuropathy and the involvement of OCS in progressive CPA, 100 mg of subcutaneous mepolizumab every four weeks was initiated; this dose was approved for severe asthma but not EGPA because mepolizumab was not approved for EGPA at that time in Japan.

Sensory impairment in the distal legs smoothly improved in six months and finally resolved with slight residual paresthesia on the bilateral planta pedis. Deteriorations in ankle dorsiflexion were also resolved (Figure 1). NCS showed a remarkable recovery of CMAP and MNCV to the reference range and a modest improvement of SNAP 27 months after starting mepolizumab (Figure 3a-c). Most of the neurological symptoms were resolved one year after the induction of mepolizumab.

In addition, the fungus ball sign collapsed in the right upper lesion on chest CT (Figure 2d), and the serum *Aspergillus* galactomannan antigen level was normalized. Prednisolone and itraconazole were discontinued seven months after the initiation of mepolizumab. Finally, mepolizumab was withdrawn 3.5 years later because of persistent clinical remission.

## Discussion

This paper reports a notable case of the successful treatment of refractory peripheral neuropathy in EGPA, which was refractory to OCS and IVIG, using the reference dose of mepolizumab for severe eosinophilic asthma. Peripheral neuropathy in EGPA is occasionally resistant to OCS, additive immunosuppressants, or IVIG. In two studies, peripheral neuropathy remained in 42% and 52% of patients with EGPA despite conventional therapies [2,14]. Additionally, the combination of OCS and CYC did not recover motor disturbance in 17% of cases or sensory impairment in the limbs in 34% of cases [16]. IVIG therapy has been reported to improve motor neuropathy to the reference level, exhibiting a ~13% or 50% increase in manual muscle testing scores [7,17]. In our case, the muscle movement disturbance was resolved and >90% CMAP was recovered in the legs (normal level) after the administration of a lower dose of mepolizumab. Additionally, symptoms of sensory disturbance and their area in the legs decreased, and ~22% of SNAP was recovered in the left sural nerve after mepolizumab administration. Based on the course of these improvements, mepolizumab remarkably contributed to the resolution of neurological distress. This mepolizumab efficacy in this study could be comparable to or superior to that of IVIG therapy for peripheral neuropathy in EGPA.

The reference dose of mepolizumab for severe eosinophilic asthma, which was lower than that for EGPA, resolved refractory peripheral neuropathy in our patient. Our case was in line with the results of an observational study reported by Kitamura et al., which showed that the reference dose of mepolizumab for EGPA (three times the dose used in our study) improved neurological symptoms in patients with EGPA [18]. Our and their cases had marked eosinophilia, but four of seven cases, including ours, did not exhibit MPO-ANCA. It was reported that EGPA patients with negativity for MPO-ANCA exhibited high numbers of eosinophils in the lumen of the epineural vessels [19], many epineural vessels occluded by intraluminal eosinophils [19], and much eosinophil infiltration and degranulation of eosinophils in the epineurium and endoneurium in the specimens of nerve biopsy, compared with the findings in those positive for MPO-ANCA [19]. The negativity for MPO-ANCA in our patient suggested that eosinophilic inflammation had similarly increased around the nerves in his lower extremities before the administration of oral prednisolone and IVIG. This might explain why the reference dose of mepolizumab for severe eosinophilic asthma improved neural damage in the patient’s lower extremities. Mepolizumab might have further inhibited the eosinophil inflammation.

In our case, the improvement in SNAPs was less than that in CMAPs, corroborating a previous study that demonstrated a lower rate of efficacy of OCS with CYC in sensory disturbance [16]. This could partly be due to the influence of resident paresthesia on the bilateral planta pedis. As the sensory symptom and its area improved more gradually than MRC, sensory neuropathy could be repaired more gradually than motor neuropathy.

In our case, mepolizumab achieved the discontinuation of OCS without any relapses and sustained remission of EGPA without OCS and additive immunosuppressants over three years. This experience corroborates a previous study that addressed the efficacy of mepolizumab on relapsing or refractory EGPA treated with conventional therapy [6]. Upon treatment with GCs alone, patients without markers of a poor prognosis obtained >90% remission during the induction phase; nevertheless, relapses were common when GCs were tapered off [11,20]. Nevertheless, EULAR recommends the use of mepolizumab as induction therapy for clinical remission in cases of recurrent or refractory EGPA that is neither life-threatening nor organ-threatening [20]. This is based on a phase III randomized double-blind placebo-controlled trial, showing that more than half of patients receiving mepolizumab experienced complete remission and a GC reduction of more than 50% [6].

Our case represents a robust example of the efficacy of mepolizumab as induction therapy for clinical remission and the sparing effect of OCS on EGPA without adverse prognostic factors. Simultaneously, the comorbid CPA improved during the reduction of the OCS dose in this case. In patients with progressive health conditions, such as chronic infection and type 2 diabetes, there is a need to consider the intensity and duration of immunosuppressive therapy. Our case suggests that the mepolizumab-sparing effect on GCs supports recovery from immunosuppressed conditions to some extent.

## Conclusions

We experienced a case of nonorgan-threatening EGPA with peripheral neuropathy resistant to conventional therapy and comorbid refractory CPA that was successfully treated with mepolizumab. Mepolizumab succeeded in inducing clinical remission that was maintained in the long term without OCS. Mepolizumab is expected to improve organ impairment due to infiltrated eosinophils in EGPA. EGPA with negativity for ANCA, characterized as the main component of organ manifestations related to eosinophilic infiltrate, is a likely indication for mepolizumab. Mepolizumab is an effective therapeutic option for refractory peripheral neuropathy and to avoid corticosteroid-related adverse events in EGPA patients.

## References

[1] Comarmond C, Pagnoux C, Khellaf M (2013). Eosinophilic granulomatosis with polyangiitis (Churg-Strauss): clinical characteristics and long-term followup of the 383 patients enrolled in the French Vasculitis Study Group cohort. Arthritis Rheum.

[2] Sada KE, Amano K, Uehara R, Yamamura M, Arimura Y, Nakamura Y, Makino H (2014). A nationwide survey on the epidemiology and clinical features of eosinophilic granulomatosis with polyangiitis (Churg-Strauss) in Japan. Mod Rheumatol.

[3] Silver J, Deb A, Packnett E, McMorrow D, Morrow C, Bogart M (2023). Characteristics and disease burden of patients with eosinophilic granulomatosis with polyangiitis initiating mepolizumab in the United States. J Clin Rheumatol.

[4] Nguyen Y, Guillevin L (2018). Eosinophilic granulomatosis with polyangiitis (Churg-Strauss). Semin Respir Crit Care Med.

[5] Morozumi S, Koike H, Tomita M (2011). Spatial distribution of nerve fiber pathology and vasculitis in microscopic polyangiitis-associated neuropathy. J Neuropathol Exp Neurol.

[6] Koike H, Sobue G (2013). Clinicopathological features of neuropathy in antineutrophil cytoplasmic antibody-associated vasculitis. Clin Exp Nephrol.

[7] Tsurikisawa N, Taniguchi M, Saito H, Himeno H, Ishibashi A, Suzuki S, Akiyama K (2004). Treatment of Churg-Strauss syndrome with high-dose intravenous immunoglobulin. Ann Allergy Asthma Immunol.

[8] Tsurikisawa N, Oshikata C, Kinoshita A, Tsuburai T, Saito H (2017). Longterm prognosis of 121 patients with eosinophilic granulomatosis with polyangiitis in Japan. J Rheumatol.

[9] Chung SA, Langford CA, Maz M (2021). 2021 American College of Rheumatology/Vasculitis Foundation guideline for the management of antineutrophil cytoplasmic antibody-associated vasculitis. Arthritis Rheumatol.

[10] Hellmich B, Sanchez-Alamo B, Schirmer JH (2023). EULAR recommendations for the management of ANCA-associated vasculitis: 2022 update. Ann Rheum Dis.

[11] Puéchal X, Pagnoux C, Baron G (2019). Non-severe eosinophilic granulomatosis with polyangiitis: long-term outcomes after remission-induction trial. Rheumatology (Oxford).

[12] Rice JB, White AG, Scarpati LM, Wan G, Nelson WW (2017). Long-term systemic corticosteroid exposure: a systematic literature review. Clin Ther.

[13] Bleecker ER, Menzies-Gow AN, Price DB (2020). Systematic literature review of systemic corticosteroid use for asthma management. Am J Respir Crit Care Med.

[14] Padoan R, Marconato M, Felicetti M (2018). Overall disability sum score for clinical assessment of neurological involvement in eosinophilic granulomatosis with polyangiitis. J Clin Rheumatol.

[15] Wechsler ME, Akuthota P, Jayne D (2017). Mepolizumab or placebo for eosinophilic granulomatosis with polyangiitis. N Engl J Med.

[16] Cho HJ, Yune S, Seok JM (2017). Clinical characteristics and treatment response of peripheral neuropathy in the presence of eosinophilic granulomatosis with polyangiitis (Churg-Strauss syndrome): experience at a single tertiary center. J Clin Neurol.

[17] Koike H, Akiyama K, Saito T, Sobue G (2015). Intravenous immunoglobulin for chronic residual peripheral neuropathy in eosinophilic granulomatosis with polyangiitis (Churg-Strauss syndrome): a multicenter, double-blind trial. J Neurol.

[18] Kitamura N, Hamaguchi M, Nishihara M (2021). The effects of mepolizumab on peripheral circulation and neurological symptoms in eosinophilic granulomatosis with polyangiitis (EGPA) patients. Allergol Int.

[19] Nishi R, Koike H, Ohyama K (2020). Differential clinicopathologic features of EGPA-associated neuropathy with and without ANCA. Neurology.

[20] Samson M, Puéchal X, Devilliers H (2013). Long-term outcomes of 118 patients with eosinophilic granulomatosis with polyangiitis (Churg-Strauss syndrome) enrolled in two prospective trials. J Autoimmun.

